# Local nonlinearity engineering of evanescent-field-interaction fiber devices embedding in black phosphorus quantum dots

**DOI:** 10.1515/nanoph-2021-0513

**Published:** 2021-10-27

**Authors:** Yuyuan Jiang, Jian Zhou, Bowen Lou, Jing Liu, Yanhua Xu, Junqing Zhao, Lei Li, Dingyuan Tang, Deyuan Shen

**Affiliations:** Jiangsu Key Laboratory of Advanced Laser Materials and Devices, Jiangsu Collaborative Innovation Center of Advanced Laser Technology and Emerging Industry, School of Physics and Electronic Engineering, Jiangsu Normal University, Xuzhou 221116, Jiangsu, China; School of Electrical and Electronic Engineering, Nanyang Technological University, Singapore 637123, Singapore

**Keywords:** black phosphorus quantum dots, evanescent field, fiber laser, local nonlinearity

## Abstract

Tapered fiber (TF) and D-shaped fiber (DF) are two types of widely investigated devices in facilitating evanescent-field interactions with external materials. Although they have been found to be particularly useful in various ultrafast regimes, to date there is still no clear or systematic investigation on their local nonlinearities as well as the exerted influences on ultrafast behaviors. Herein, we present such thorough investigation through local nonlinearity engineering on TF and then in contrast with a DF as a reference. Optically deposited black phosphorus quantum dots (BPQDs) are used for saturable absorption. The nanometer-scale extremely small sizes of the BPQDs helpfully eliminate size-induced uncertainties or distortions during both device fabrication and the latter light–matter interaction. For the TF, in the experiment, it is found that the local nonlinear effect starts to be rather appreciable as the TF shrinks to a moderate thickness. Remarkably in comparison, the local nonlinearity of the DF itself can even be neglected reasonably, but after coating with BPQDs, it possesses a much larger modulation depth than any of the used BPQDs-coated TFs with different thicknesses/lengths. Further, we theoretically analyze the related locally nonlinear effects and reveal, for the first time, the direct origin of saturable absorption with evanescent-field-based general structures.

## 1 Introduction

Since the successful application of carbon nanotubes (CNTs) and graphene as saturable absorbing materials in various ultrafast laser systems, especially fiber lasers [1], [2], [3], [4], [5], [6], [7], [8], [9], a wide variety of micro/nanomaterials have been explored and fabricated as saturable absorbers (SAs). Those include nanoscale metal particles [10, 11], topological insulators [12], [13], [14], MXene [15, 16], transition metal dichalcogenides [17], [18], [19], black phosphorus [20], [21], [22], [23], [24], [25], etc. In principle, they all can be applied in both bulk solid-state and fiber lasers. However, considering that substantial care and effort are typically required in the alignment with typical bulk systems, the greatly simplified fiber lasers have been most frequently used as the testbeds to investigate the saturable absorbing properties of these materials.

To incorporate micro/nanomaterials into fiber laser cavities, many different approaches can be implemented. Dissipating them into a polymer film patch [1], [2], [3], [4], [5], [6], [7], [8], [9], [10] and directly depositing them onto a reflective mirror/transmissive window [11], [12, 26], [27] are the earliest and most commonly adopted approaches, mainly due to the simplicity in fabrication and then the ease to transfer. However, one limitation with these approaches is that they can only be used with low optical powers. This is because that such a type of thin film has a very low laser-induced damage threshold (LIDT) when exposed directly to the intensive light tightly confined within a fiber core, typically less than 100 μm^2^ in the mode-field area (MFA).

To overcome the low LIDT issue with film-type SA, an attractive alternative is coating these materials to some evanescent-field-coupling fiber devices. In this way, the light–matter interaction is based on an evanescent field rather than the guided light propagating within the fiber core. Comparatively, the evanescent-field interaction is an indirect and long-range process, which greatly reduces the light intensity accessing the material and, meanwhile, still enables adequate absorption through a distributed manner. It has been verified that such a type of device can substantially improve the LIDT in contrast with typical thin films.

The commonly used fiber devices that enable evanescent field interaction include tapered fiber (TF) [28], [29], [30], [31], [32], [33], [34], side-polished D-shaped fiber (DF) [35], [36], [37], [38], [39], [40], [41], [42], [43], photonic crystal fiber (PCF) [44], [45], [46], [47], [48], [49], [50], [51], etc. All these devices show significant enhancement in power handling and can preserve the all-fiber structure of the laser cavity. Among them, TF and DF rank the two most frequently used devices. Besides their ease in fabrication, they can be loss-freely spliced with other commercial fiber pieces.

However, to date, no clear demonstration can be found on what effects might be induced by the local properties of these two widely investigated fiber devices, especially regarding their local nonlinearities and the physical mechanisms of saturable absorption in the evanescent-field manner. To address these, in this paper, we intend to investigate thoroughly on both the TF and DF devices. It is achieved by varying sizes of TF and DF and then comparing their characteristics in nonlinear absorption and passive mode-locking. To reduce the uncertainties during the investigation, a type of ultrasmall black phosphorus quantum dots (BPQDs) is used as the saturable absorbing material. Although many two-dimensional (2D) materials have been investigated widely for applications in ultrashort pulse generation [52], BPQDs still hold some recognized outstanding optical properties. As noted, three of them are particularly remarkable when they are used for ultrashort pulse generation. Firstly, BPQDs have verified much lower saturable intensity, larger modulation depth, and lower saturation intensity than BP nanosheets and other 2D-layered BP materials [53]. Secondly, BP has an ultrafast recovery time which reaches 24 ± 2 fs, faster than other commonly investigated materials [54]. Thirdly, the single-layer absorbance of BP can reach ∼2.8, which is greater than that of graphene ∼2.3% [55].

## 2 Fabrication of BPQDs

**Figure 1:**
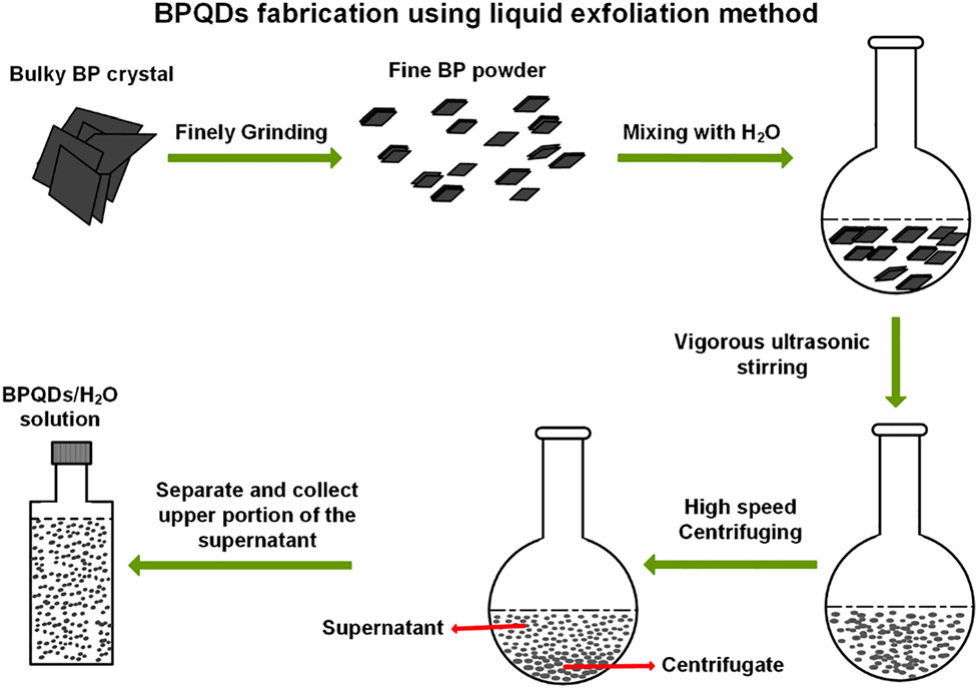
Fabrication process of BPQDs using liquid exfoliation method.

Figure 1 schematically shows the fabrication process of our used BPQDs, which is based on a typical liquid exfoliation method and completed in Nanjing XFNANO Materials Tech Co., Ltd. The process is also similar to other early reports [53, 56], [57] except for some minor differences in specific procedures. It should be noted that, here, distilled water (H_2_O) is used as the solvent during the fabrication process. One consideration is that the finely ground BP powders, as well as the finally fabricated BPQDs, can be more preferably dispersed in distilled water than other usually used solvents, like *N*-methyl-2-pyrrolidone [53]. This should mainly benefit from the polarity of the H_2_O molecule. Another consideration is that the distilled water is easy to remove after depositing to the TF and DF devices.

**Figure 2:**
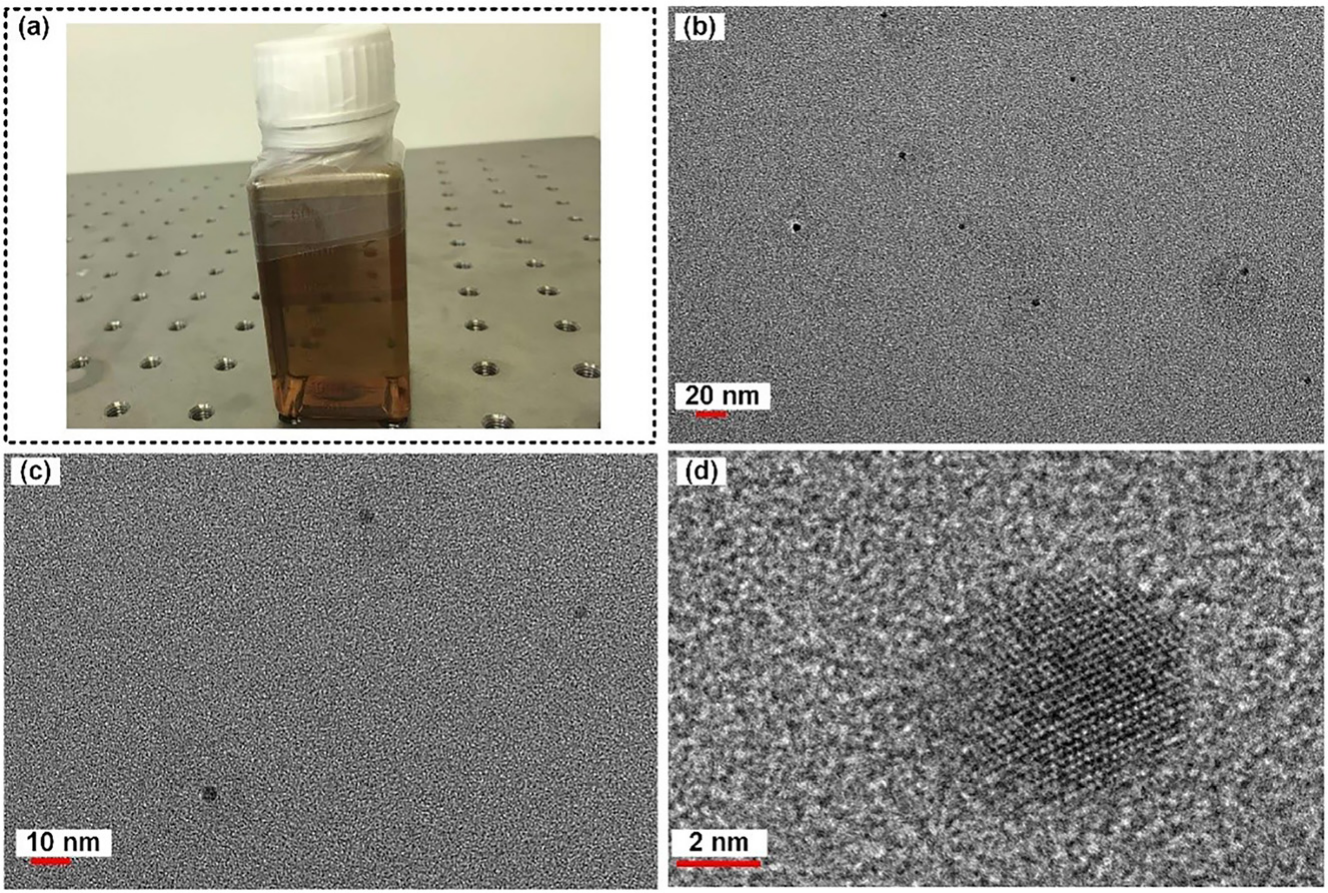
(a) BPQDS/H_2_O solution; (b) and (c) different scales of TEM; (d) HRTEM.

Figure 2(a) shows the finally fabricated BPQDS/H_2_O solution sealed in a glass bottle. Transmission electron microscopy (TEM) is employed to characterize the morphology of BPQDs. The TEM images in Figure 2(b) and (c) evidence the existence of ultrasmall BPQDs and also reveal that different BPQDs vary little in size, all having diameters less than 5 nm. Figure 2(d) shows a high-resolution TEM (HRTEM) image of the BPQDs. Regular lattice fringes can be evidently seen, indicating that such a BPQD consists of a single layer of atoms. The extremely small sizes of the BPQDs can eliminate the size-related uncertainties [58].

## 3 Experimental details of BPQDs-deposited microfiber devices in ultrafast fiber lasers and related discussions

### 3.1 BPQDs-coated TFs for mode-locked fiber lasers

**Figure 3:**
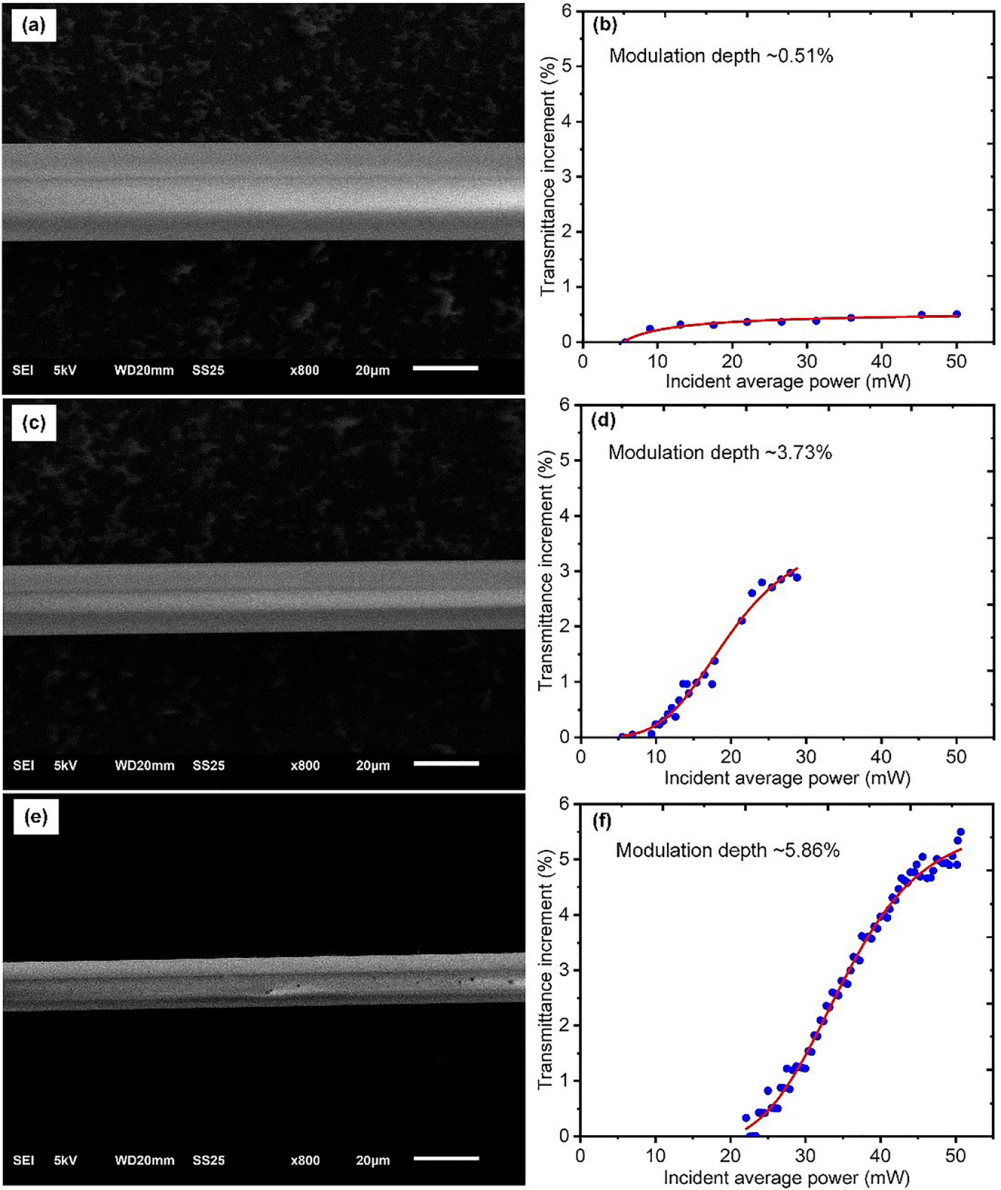
(a), (c) and (e) Three different house-drawn fiber tapers with taper lengths of ∼20 mm, ∼25 mm, and ∼30 mm, respectively; (b), (d), and (f) the corresponding saturable absorbing characteristics after coating BPQDs.

All the TFs here are drawn with the flame-brushing method by using a fiber tapering workstation (IPCS-5000, Idealphotonics Inc.). Figure 3(a), (c), and (e) show scanning electron microscopy images around the waists of three differently drawn TFs that are used in the following experiments. As a measurement, the waist diameters are ∼30, ∼18.5, and ∼15 μm, corresponding to taper lengths of ∼20, ∼25, and ∼30 mm, respectively.

The previously fabricated BPQDs are optically deposited onto these TF and DF devices to enable evanescent-field interactions. Moreover, to ensure certainty in the investigation the used optical deposition parameters are roughly the same, including the depositing duration, launched light intensity in each fiber device, concentration of the used BPQDs/H_2_O solution, etc. The detailed process is described below.

The BPQDs are coated onto the TFs and DF by adopting exactly the same procedure. At first, ∼3 mL BPQDs/H_2_O solution is prepared. During all the processes, the used solution samples exhibit the same concentration of BPQDs. Following, a part of a TF or DF is immersed into the solution. All the immersed parts of the TFs and DF roughly share the same length. Then, ∼105 mW laser light at 980 nm is launched into the TF or DF for optical deposition. The BPQDs coated TF or DF is finally completed after depositing for ∼1 h.

All the saturable absorption characteristics through this paper are measured by using a house-built ultrafast erbium-doped fiber (EDF) laser with power boosting in an EDF amplifier (EDFA). The seeding EDF laser produces mode-locked pulses with duration of ∼1 ps at ∼25 MHz repetition rate. The average power from the seeding EDF is ∼2 mW, which can be amplified to >50 mW in the EDFA. According to our measurements, roughly to say, the thinner the TF is, the larger the acquired modulation depth. Figure 3(b), (d), and (f) plot the measured absorbing characteristics of the BPQDs-coated TFs with taper lengths of ∼20 mm, ∼25 mm, and ∼30 mm long, respectively. For convenience in comparison, here we plot the transmittance increment as a function of incident average power and use the same coordinate scales. According to nonlinear fit, i.e., the red curves in Figure 3(b), (d), and (f), their modulation depths are ∼0.51%, ∼3.73%, and ∼5.86%, respectively. As measurement (not shown in the figure), the nonsaturable loss also increases from ∼14.74 to ∼19.53% as the taper lengthens from ∼20 to ∼30 mm.

It should be noted that both the BPQDs in H_2_O and the BPQDs coating on fiber devices can be oxidized gradually [56]. In the experiment, we notice that the BPQDs can maintain their saturable absorption properties for roughly one week when preserved as BPQDs/H_2_O solution. This is in agreement with the results in Ref. [59]. However, the saturable absorption effects will nearly disappear in roughly three days when the BPQDs are exposed to air. Since here our main purpose is to investigate the local nonlinearities of the fiber devices by taking advantage of the characteristic uniformity of BPQDs, we only keep the BPQDs/H_2_O solution and BPQDs coated TF or DF in dark sealed boxes. Despite that, no other particular protective measures have been taken. Somehow this can ensure that, during our present experiment and tests, no noticeable degradation in saturable absorption can be observed. The seeking of reliable protective measures would be one of our future efforts.

**Figure 4:**
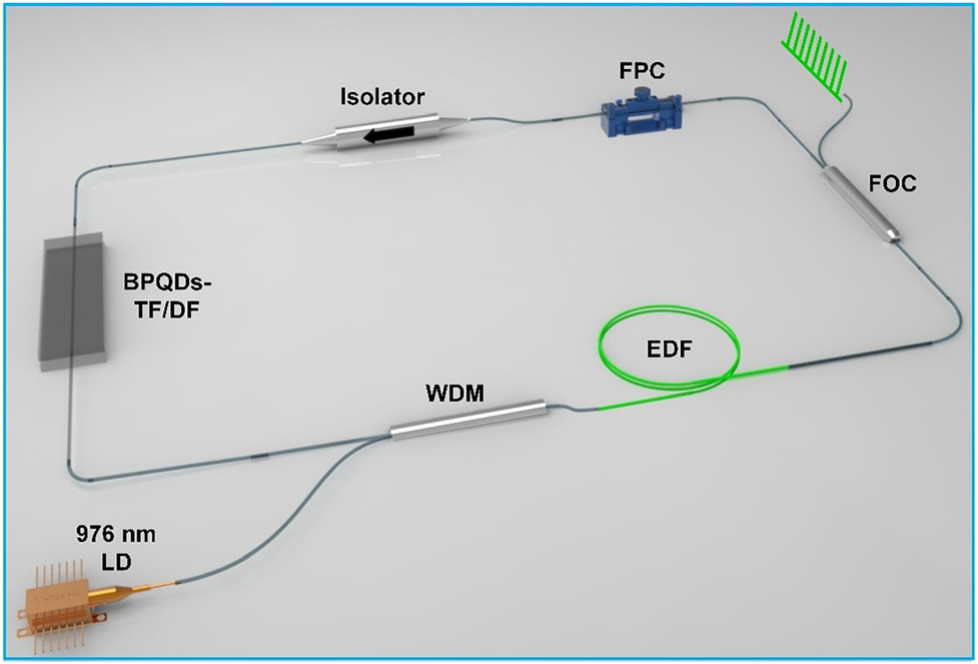
Schematic EDF laser setup with the incorporation of BPQDs-coated TF/DF.

Figure 4 shows a schematic setup of the used EDF laser for testing the mode-locking characteristics by using the BPQDs-coated TFs as well as the latter BPQDs-coated DF. The EDF laser exhibits a typical ring-cavity configuration. A single-mode laser diode with a central wavelength at ∼976 nm is used as the pumping source. The pump light is coupled into a piece of ∼0.8 m long EDF (RightWave^®^ EDF80, OFS Fitel, LLC.) via a wavelength division multiplexer. The EDF has a core numerical aperture of ∼0.28, mode field diameter of ∼4.3 μm, peak absorption at 1530 nm of ∼80 dB/m, and dispersion at 1550 nm of ∼−48 ps/(nm·km). Such a piece of EDF is mainly used to provide appropriate laser gain. Meanwhile, it can partially compensate for the net anomalous cavity dispersion since it exhibits normal dispersion around the lasing wavelength. A fiber optical coupler is used to couple ∼20% power out of the cavity as the output. An in-line fiber polarization controller is used to manipulate the light polarization state via tuning the local fiber birefringence. A fiber isolator is used to maintain the unidirectional propagation of the signal light in the fiber laser cavity. The other connecting fibers in the cavity are all the same type of standard single-mode fiber (SMF, SMF-28e, Corning Incorporated).

**Figure 5:**
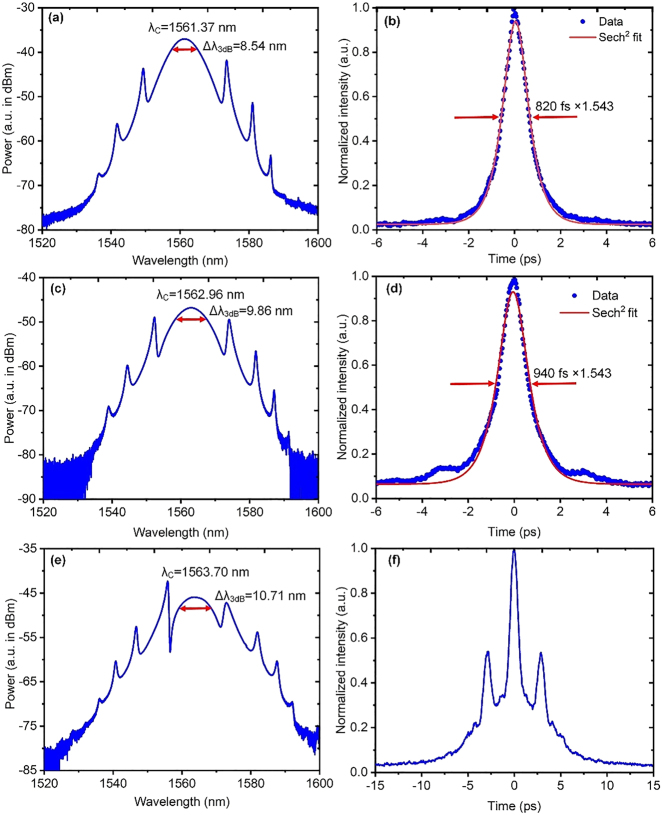
Spectra and AC traces when the EDF laser was mode-locked by using three different lengths of BPQDs-coated TFs: (a) and (b) ∼20 mm; (c) and (d) ∼25 mm; (e) and (f) ∼30 mm.

Figure 5 comparatively shows the mode-locking characteristics when the aforementioned three different sizes of BPQDs-coated TFs are incorporated in the EDF laser, respectively. The pump thresholds are 50, 50, and 60 mW, respectively. As seen from Figure 5(a), (c) and (e), the produced 3-dB spectral bandwidth widens significantly as the TF becomes thinner and longer. All the spectra through this paper are measured by using the same optical spectrum analyzer (OSA, AQ6370C, Yokogawa Test &Measurement Co.) with the resolution setting at 0.02 nm. When the used TF is ∼20 mm long, the EDF laser produces a perfect single soliton, of which the spectrum contains three pairs of symmetrical Kelly sidebands as seen in Figure 5(a). The emitted central wavelength and 3-dB spectral bandwidths are ∼1561.37 and ∼8.54 nm, respectively. Figure 5(b) plots the corresponding autocorrelation (AC) trace, giving a Sech^2^-fitted pulse width of ∼820 fs. All the AC traces through this paper are measured by using the same autocorrelator (FR-103HS, Femtochrome, Inc.) connecting to the same digital oscilloscope (DSO-X 3034A, Keysight Technologies).

When the used TF is ∼25 mm long, the EDF laser can still produce a typical soliton that exhibits symmetrical Kelly sidebands on the spectrum, as seen in Figure 5(c). But, now the 3-dB spectral bandwidth broadens to ∼9.86 nm. This should result from the strengthened self-phase modulation (SPM) effect. This is mainly determined by the enhanced local nonlinearity with the present TF considering that all other cavity parameters are completely the same as before.

The corresponding AC trace now has a Sech^2^-fitted width of ∼940 fs, as seen in Figure 5(d), larger than that in Figure 5(b). Meanwhile, the AC trace exhibits some pedestal. Both features with the AC trace indicate that there should exist some nonlinear chirp with present soliton again due to the enhanced local nonlinearity by using the ∼25 mm TF. This nonlinear chirp cannot be fully compensated, and, as a consequence, the produced soliton deviates more from the Fourier-transform limit, i.e., a longer soliton duration, and the time-bandwidth product (TBP) also becomes larger than ∼0.315. Another effect leading to the longer pulse duration with the ∼25 mm TF should be that this TF has a slightly larger anomalous dispersion than that of the ∼20 mm TF [60, 61]. This results in a larger net anomalous cavity dispersion and thus a longer soliton duration. It is still possible that this TF can induce some larger third-order dispersion, which can also result partially in the formation of a soliton pedestal and longer soliton duration.

As the 30-mm long BPQDs-coated TF is used, the output spectrum further broadens to ∼10.71 nm, as seen in Figure 5(e). However, now the emitted pulse from the EDF laser is not perfect but a broken soliton, of which the measured AC trace breaks into three parts as seen in Figure 5(f), indicating that now the generated pulse should be a type of higher-order soliton [62].

It is further noticed that, apart from the obtained higher-order soliton characterized as Figure 5(e) and (f), other mode-locking states can also be observed by manipulating the intra-cavity polarization state when the same ∼30 mm long BPQDs-coated TF is used. Among them, there are two emission states that are relatively stable. One is a bound-state of two solitons and the other is a type of completely split dual solitons.

**Figure 6:**
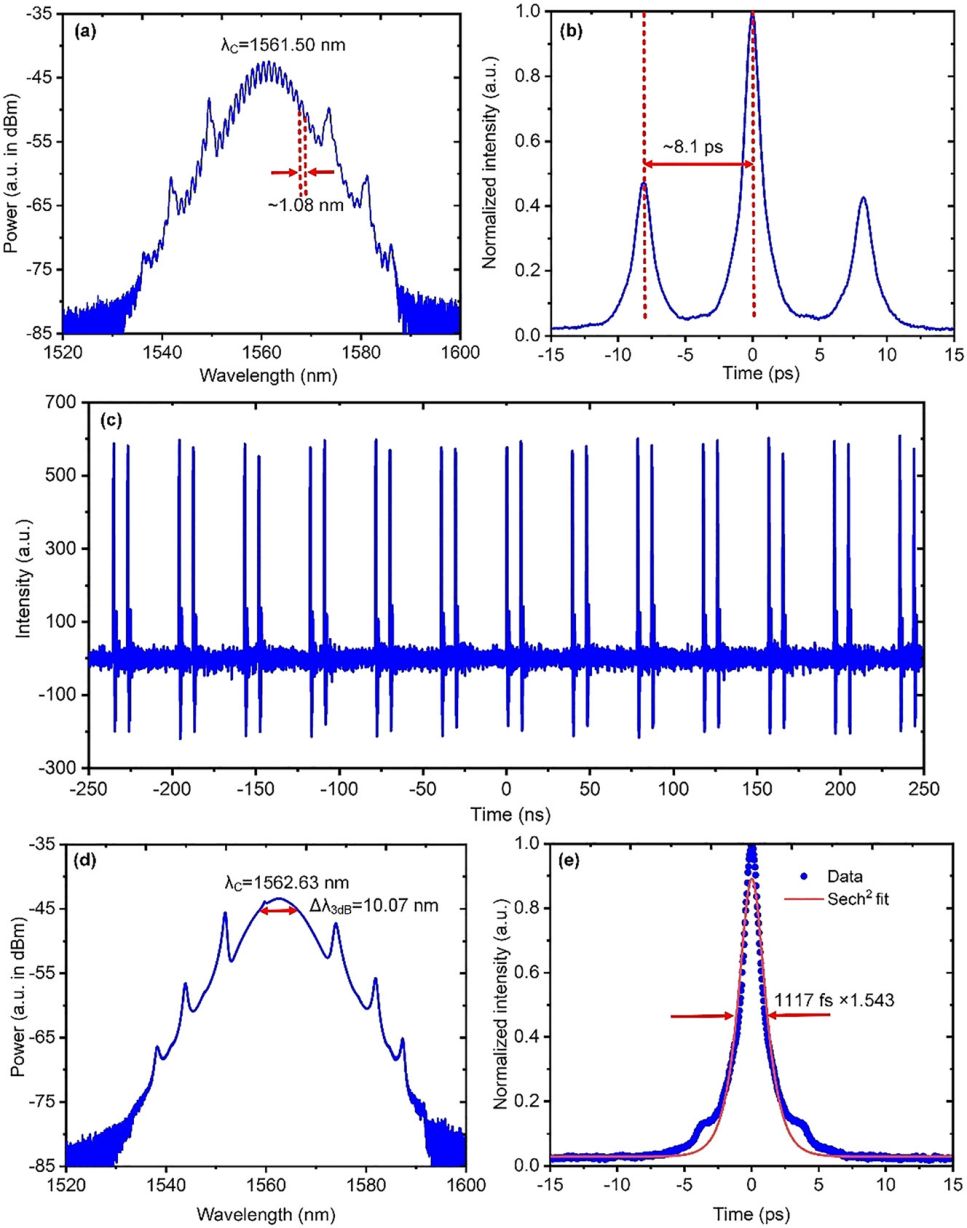
Bound-state and completely split solitons by using the same 30 mm long BPQDs-coated TF. Bound-state solitons: (a) output spectrum; (b) AC trace of the bound-state soliton. Completely split solitons: (c) a typical pulse train with 500-ns span; (d) output spectrum; (e) AC trace with Sech^2^-fit.

As for the bound-state solitons, there appears a type of periodically modulating pattern on the spectrum as seen in Figure 6(a), as a result of spectral interference. Figure 6(b) plots the corresponding AC trace, showing a typical feature of two solitons coherently bounded together [63, 64]. Thus, this further verifies that the modulating pattern in Figure 6(a) directly results from the interference between these two temporally bounded and overlapped solitons. The modulation period of ∼1.08 nm and the temporal separation between the two solitons of ∼8.1 ps just satisfy the general relation of typical bound-state solitons [65],

(1) $$\Delta \tau =\lambda^{2}/\left(c\Delta \lambda \right)\text{,}$$

where Δ*τ* is the pulse separation, *λ* the central wavelength, *c* the light propagation speed in a vacuum, and Δ*λ* the spectral modulation period.

Figure 6(c) through (e) show output characteristics with the completely split dual-soliton state which is achieved by using a higher pump power of ∼75 mW. Figure 6(c) shows a typical pulse train. Through this paper, all the pulse trains are detected by using the same InGaAs biased photodetector (PD) (DET01CFC/M, Thorlabs, Inc.), and then recorded by using the same digital oscilloscope (DSO9104A, Agilent Technologies, Inc.). As seen in Figure 6(c), now the two solitons split completely, with no bounding energy to associate them temporally. Even that, they can still maintain a stable state with a fixed temporal separation of ∼5.9 ns that is long enough to be detected and resolved by using the aforementioned PD (rise time <1 ns). The spectrum, as shown in Figure 6(d), still has a typical solitonic profile with symmetrical Kelly sidebands, but becomes narrower than that in Figure 5. This is due to that split solitons have much lower peak powers than that of the unbroken higher-order soliton, resulting in a relatively weaker SPM-induced spectral broadening effect. The appeared significant pedestals on the AC trace, as seen in Figure 6(e), indicate that there should also be some uncompensated nonlinear chirp with the solitons, which is similar to that in Figure 5(d).

The formation of higher-order soliton and the origins of the other different states can be understood as follows.

The net anomalous dispersion of the laser cavity enables the soliton formation when the SPM and group velocity dispersion (GVD) can maintain their balance. However, if the intra-cavity nonlinear effect is too strong, the SPM effect will dominate the soliton evolution initially. In our case it is just true considering that the ∼20 or ∼25 mm TF has been replaced by a thinner and longer TF (∼30 mm), comparatively resulting in an abrupt enhancement of the local nonlinearity. Although the SPM can dominate the initial stage of the soliton evolution, the GVD will very soon play its role through contracting the soliton [62], consequently leading to a significantly higher peak-power pulse which is typically called higher-order soliton. It can be seen that the main peak in Figure 5(f) is clearly much narrower than the AC traces in Figure 5(b) and (d). The constantly delaying interplay between the SPM and GVD will lead to temporally and spectrally periodic evolution of the higher-order soliton. As noted, in our case the soliton order should be *N* = 2 considering that the peak number *m* of the autocorrelation (AC) trace [Figure 5(f)] satisfies the relation

(2) m=2N−1.

It should be noted that although all the fiber pieces and devices in the laser cavity are polarization-independent as specified, there is still some possibility that a certain degree of polarization dependence exists considering the defects during fabrications. Thus, through manipulating the intra-cavity polarization state the intra-cavity soliton will experience different amounts of polarization-dependent loss, residual birefringence, nonlinear phase shift, GVD, as well as some other higher-order or even unknown effects. That is why we can obtain the other two different soliton states employing polarization manipulation, i.e., a bound-state of two solitons and a type of completely split dual solitons.

There are two main differences between the higher-order soliton and bound-state soliton although both of them show no pulse splitting on the oscilloscope. At first, the higher-order soliton is still a single soliton, but the bound-state soliton contains actually two closely and coherently existing solitons which can be revealed by using an autocorrelator. Secondly, the evolution of higher-order soliton exhibits intrinsic periodicity during circulating in the fiber ring cavity, but the solitons in bound-state can always maintain their phase relation during propagation due to the attractive and repulsive force balance during their coherent interaction.

### 3.2 BPQDs-coated DF for mode-locked fiber laser

**Figure 7:**
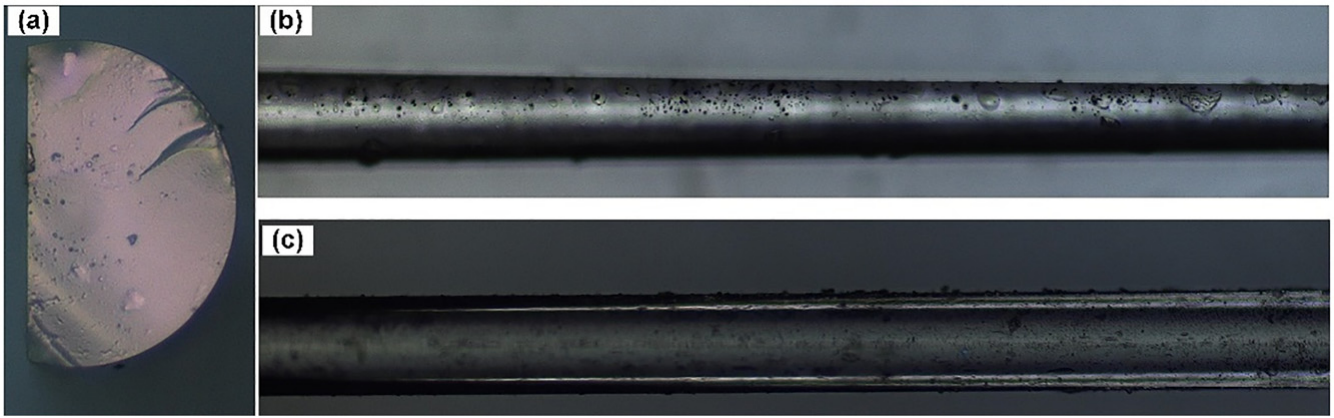
(a) Cross-section of the DF. (b) Side view and (c) top view of the transition region from the unpolished to the polished part of the DF.

Figure 7 shows the images of the DF viewed from different directions by using an optical microscope. Figure 7(a) shows the cross-section of the D-shaped portion, highly resembling the capital letter D. Figure 7(b) and (c) show the transition region from the unpolished to the polished part of the DF viewing from side and top directions, respectively.

**Figure 8:**
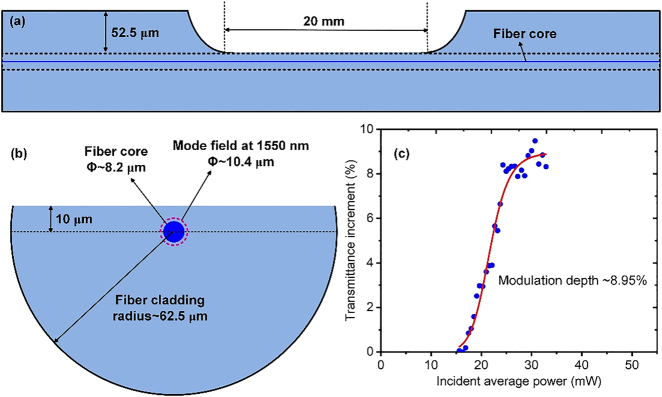
Schematic (a) side section and (b) cross-section of the DF. (c) Measured nonlinear absorbing characteristics of the BPQDs-coated DF.

To quantitatively understand the waveguiding characteristics of the DF, we schematically illustrate the side and cross-sections as seen in Figure 8(a) and (b), respectively. As shown in Figure 8(a), the D-shaped portion is ∼20 mm long and ∼52.5 μm deep. Considering that the fiber radius is ∼62.5 μm, the surface of the D-shaped portion to the fiber core center is ∼10 μm, which is greater than the fiber core radius of ∼4.1 μm and even greater than the mode field radius of ∼5.2 μm at a wavelength of 1550 nm. Figure 8(b) clearly illustrates their numerical relations.

Figure 8(c) plots the nonlinear absorbing characteristics of the BPQDs-coated DF, where the used coordinate scale of the horizontal axis is the same as those in Figure 3(b), (d), and (f), for clarity in comparison. Now the nonsaturable loss is measured to be only ∼0.88% (not shown in the figure), implying that the BPQDs-coated DF is roughly loss-free for an incident pulse with peak power large enough but still below LIDT. This is also understandable, considering that the surface of the D-shaped part is still distance-away from the fiber core (∼5.9 μm) and the induced change on the guided wave is consequently weak during the evanescent wave interaction with the coated BPQDs. The modulation depth of the BPQDs-coated DF is ∼8.95% via nonlinear fit to the measured data. This, interestingly, is evidently greater than any of the previously used BPQDs-coated TFs.

**Figure 9:**
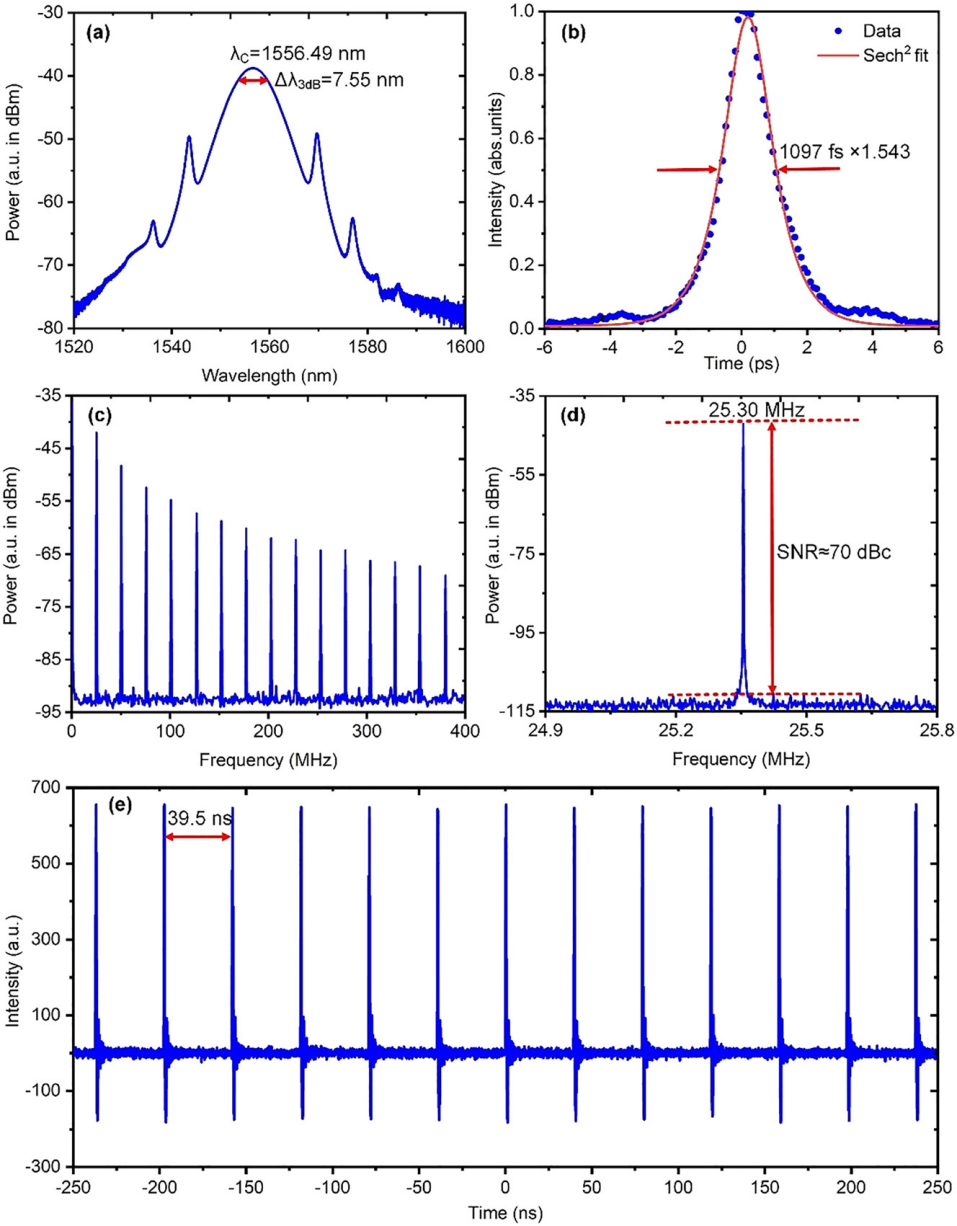
Mode-locked characteristics when incorporating the BPQDs-coated DF into the EDF laser. (a) Output spectrum. (b) AC trace with Sech^2^-fit. (c) RF trace with a span of 0–400 MHz and RBW of 1 kHz. (d) RF trace around the fundamental repetition rate with an RBW of 10 Hz. (e) A typical pulse train with a 500 ns span.

Figure 9 shows the mode-locking characteristics by using the BPQDs-coated DF as the SA in the same EDF laser. Figure 9(a) plots the output spectrum with a central wavelength of ∼1556.49 nm and 3-dB spectral bandwidth of ∼7.55 nm. It is noted that the central wavelength becomes shorter and spectral bandwidth becomes narrower compared to the previously obtained spectra mode-locked by using the BPQDs-coated TFs. The shorter wavelength indicates that the DF has a smaller insertion loss than the TFs. The reduction of insertion loss, equivalent to the weakening of reabsorption at the shorter wavelengths, results in a net gain closer to the gain center of erbium ions around 1550 nm. This can also be verified by the improved average power with the same pump power compared to that using TFs, as seen in Table 1. The comparatively narrower spectrum indicates that the SPM-induced spectral broadening is also weaker. This is mainly caused by the weak local nonlinearity of the DF. In fact, the DF varies little from other parts of the SMF-28e fiber as for local nonlinearity, considering that the side-polished surface of the flat surface of the DF does not reach the fiber core and even does not reach the MFA of the propagating light wave. Thus, further considering the short length of the used DF, its local nonlinearity can even be neglected. In this sense, using the DF as a reference is reasonable to identify how much local nonlinearity is introduced by using other microfiber devices, such as the TFs that we just discussed.

**Table 1:** Power characteristics with different microfiber devices and operation regimes.

Microfiber device	Pulse type	Pump power (mW)	Output average power (mW)
BPQDs/TF∼20 mm	Single soliton	50	1.95
BPQDs/TF∼25 mm	Single soliton	50	1.663
BPQDs/TF∼30 mm	High-order soliton	60	1.55
BPQDs/TF∼30 mm	Bound-state	60	1.45
BPQDs/TF∼30 mm	Split dual-solitons	75	1.5
BPQDs/DF∼20 mm	Single soliton	50	2

Figure 9(b) plots the corresponding AC trace with Sech^2^-fit, giving a pulse width of ∼1097 fs, which is slightly longer than that of the single soliton obtained by using TFs. This is straightforward considering that the spectrum is narrower in contrast and meanwhile their TBPs should be both close to the Fourier-transform limit value of ∼0.315 for an ideal soliton. We further use the aforementioned PD and an RF spectrum analyzer (N9320B, Agilent, Inc.) to measure the related RF characteristics. The RF traces in Figure 9(c) and (d) indicate that the EDF laser operates in the single soliton regime. The signal-to-noise ratio of the RF spectrum around the fundamental repetition rate reaches 70 dB, verifying the high stability of the mode-locked operation. These can also be ensured by the pulse train plotted in Figure 9(e).

### 3.3 Discussion on the TF and DF related nonlinearities and modulation depths

As aforementioned, the local nonlinearity of the DF can be neglected considering that the D-shaped portion only has a length of 20 mm and its core size still equals that of the original SMF. Thus, there should be no noticeable contribution of spectral broadening due to the local nonlinearity of the DF. If there is any, it should all result from the accumulative nonlinearities from other cavity parts rather than the DF itself.

However, comparatively, the local nonlinearities of the TFs cannot be neglected again, which can indeed induce new spectral components, resulting in significant spectral broadening. This further indicates that the light is still mainly confined within the tapered core region, considering that the TF diameter (∼30 μm) is still much greater than the core diameter of SMF (∼8.2 μm) before tapering. Otherwise, there should be no noticeable local nonlinear effects if the MFA can match the cross-section of the TF.

If only considering the SPM effect, fiber nonlinearity can be generally characterized by using the nonlinear phase shift $\phi_{\text{NL}}$ . In fact, this is a typical case for any mode-locked fiber laser where the intra-cavity pulse is still too weak to induce other nonlinear effects, such as stimulated Raman scattering, self-steepening effect, etc. As definition [62], the nonlinear phase shift

(3) $$\phi_{\text{NL}}=\gamma P_{0}L\text{,}$$

where, *γ* is the fiber nonlinear parameter, *P*_0_ is the pulse peak power, and *L* is the used fiber length if its loss is negligible. *γ* is defined as

(4) $$\gamma =\frac{2\pi n_{2}}{\lambda A_{\text{eff}}}\text{,}$$

where *n*_2_ is the fiber nonlinear-index coefficient, *λ* is the pulse center wavelength, and *A*_eff_ is the effective MFA. From Eqs. (3) and (4), it can be seen that, despite the intrinsic material property of the fiber and the pulse parameters, $\phi_{\text{NL}}$ directly relates to *L* and *A*_eff_ as

(5) $$\phi_{\text{NL}}\propto L/A_{\text{eff}}.$$

Based on Eq. (5), the ratio between the local $\phi_{\text{NL}}$ induced by the D-shaped portion of the DF and tapering regions of the three TFs can be calculated. For simplicity, here we use the core area to replace *A*_eff_ as a rough calculation. Thus,

(6) $$\phi_{\text{NL}}\left(\text{DF}∼20\right):\phi_{\text{NL}}\left(\text{TF}∼20\right):\phi_{\text{NL}}\left(\text{TF}∼25\right):\phi_{\text{NL}}\left(\text{TF}∼30\right)=1:17.36:57.08:104.18\text{.}$$

This clearly reveals why the TFs can result in much more significant nonlinear effects than the DF.

Calculation on geometric dimensions of the DF and TFs can further help to understand why the modulation depth of the DF is greater than that of any of the used TFs. As seen in Figure 9(b), the distance from the D-shaped flat surface to the nearest edge of the fiber core is

(7) dDF=(10−12×8.2)μm=5.9 μm

We assume that the clad-to-core ratio of the SMF remains roughly unchanged after tapering. Thus, after tapering the distance from the cladding surface to the core surface is

(8) $$d_{\text{TF}}=r_{\text{TF},\text{ clad}}-r_{\text{TF},\text{ core}}=\left(1-\frac{r_{\text{SMF},\text{ core}}}{r_{\text{SMF},\text{ clad}}}\right)r_{\text{TF},\text{ clad}}$$

For the TFs with lengths of ∼20, ∼25, and ∼25 mm, corresponding to waist diameters of ∼30, ∼18.5, and ∼15 μm, *d*_TF_ can be calculated as ∼14.0, ∼8.6, and ∼7.0 μm, respectively, by using Eq. (8). Thus, each of the three TFs has a greater *d*_TF_ than *d*_DF_. Thus, the intensity of the evanescent field reaching the DF surface should be stronger than that reaching any of the TF surfaces, i.e., closer to the saturation value of the coated BPQDs. That is why the DF has a larger modulation depth than any of the TFs.

### 3.4 Origin analysis of the saturable absorption with general evanescent-field-based structures

**Figure 10:**
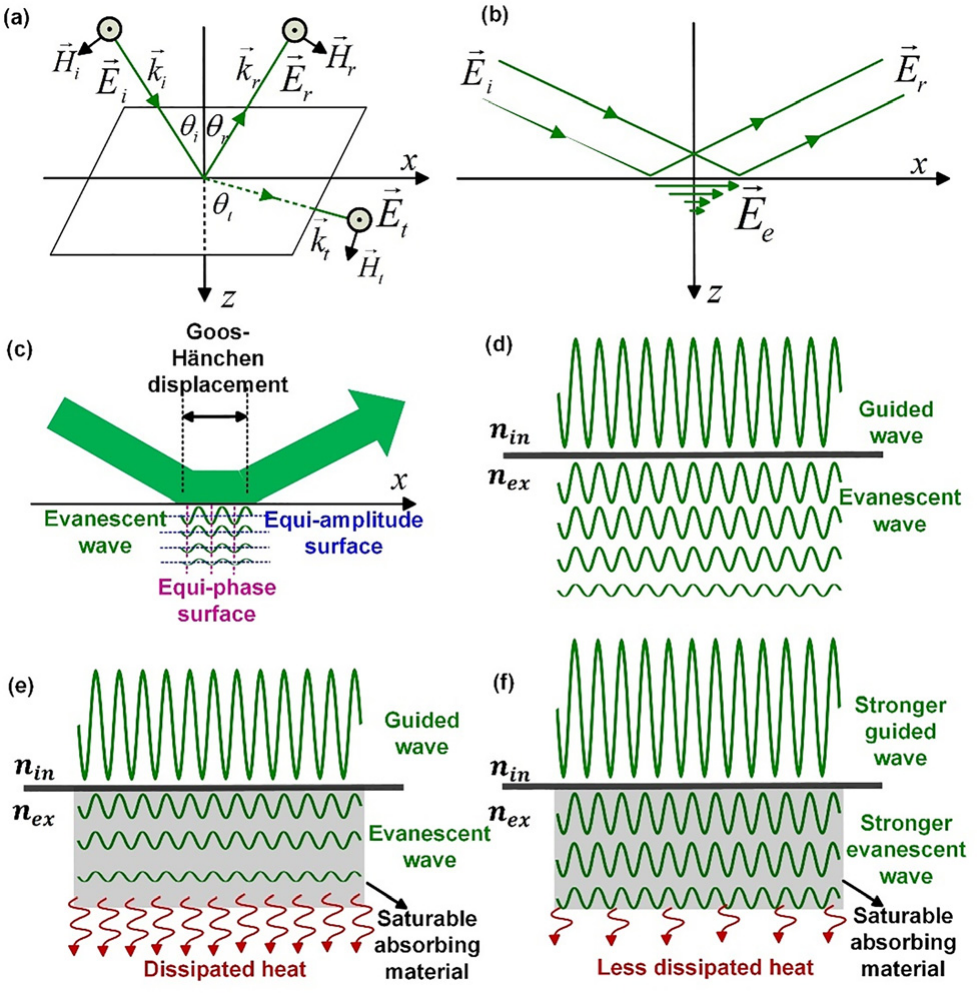
Evanescent field characteristics at the vicinity of a waveguiding interface. (a) Reflection and refraction by a general planar boundary when the light beam incident from a medium with a higher refractive index to a medium with a lower refractive index. (b) Total reflection in the incidence plane. (c) Detailed characteristics near the boundary with evanescent wave generation and Goos–Hänchen displacement. (d) Free-propagating characteristics of both the guided wave and evanescent wave. Waveguiding characteristics with externally coated saturable absorbing material when the guided wave is (e) weak and (f) strong, respectively.

To further understand the mechanisms behind the saturable absorption with evanescent-field-based structures, we start from the general case when a beam of lightwave incidents from a medium with a higher refractive index to one with a lower refractive index, as seen in Figure 10(a). For simplicity, we assume that the incident light wave is monochromatic and planar. We use ${\vec{E}}_{t}$ to represent the light field transmitting into the second medium with a lower refractive index, which can be written in the general form as

(9) $${\vec{E}}_{t}={\vec{E}}_{t0}\exp[i({\vec{k}}_{t}\cdot \vec{r}-\omega t)]\text{,}$$

where ${\vec{E}}_{t0}$ is the transmitted light field vector as a function of position, ${\vec{k}}_{t}$ the transmitted wave vector, $\vec{r}$ the position vector, and *ω* the angular frequency of the light wave. ${\vec{k}}_{t}\cdot \vec{r}-\omega t$ , as a whole, represents the phase term. Similarly, the light field prior to entering the second medium can be noted as ${\vec{E}}_{i}={\vec{E}}_{i0}\exp[i({\vec{k}}_{i}\cdot \vec{r}-\omega t)]\text{.}$

The angle of refraction *θ*_*t*_ directly relates to the magnitude *k*_*t*_ of the transmitted wave vector ${\vec{k}}_{t}$ and its *x* component *k*_*tx*_ as

(10) $$\sin\theta_{t}=\frac{k_{tx}}{k_{t}}\text{.}$$

We use *n*_int_ and *n*_ext_ to denote the refractive indexes of the internal medium and external medium of the waveguide, respectively. Thus, the magnitudes of the incident, reflected, and refracted wave vectors can be further written as

(11) $$k_{i}=k_{r}=\frac{n_{\text{int}}\omega }{c},\;k_{t}=\frac{n_{\text{ext}}\omega }{c}\text{.}$$

Based on the law of refraction [66],

(12) $$n_{\text{int}}\sin\theta_{i}=n_{\text{ext}}\sin\theta_{t}.$$

*z* component of the transmitted wave vector, i.e.,

(13) $$k_{tz}={\left(k_{t}^{2}-k_{tx}^{2}\right)}^{\frac{1}{2}}\text{,}$$

Can then be written as

(14) $$k_{tz}=\frac{\omega n_{\text{int}}}{c}{\left[{\left(\frac{n_{\text{ext}}}{n_{\text{int}}}\right)}^{2}-\sin^{2}\;\theta_{i}\right]}^{\frac{1}{2}}\text{.}$$

The critical angle *θ*_*c*_ for total reflection satisfies

(15) $$\sin\theta_{c}=\frac{n_{\text{ext}}}{n_{\text{int}}}.$$

Thus, Eq. (14) can be written as

(16) $$k_{tz}=\frac{\omega n_{\text{int}}}{c}{\left(\sin^{2}\;\theta_{c}-\sin^{2}\;\theta_{i}\right)}^{\frac{1}{2}}=i\frac{\omega n_{\text{int}}}{c}{\left(\sin^{2}\;\theta_{i}-\sin^{2}\;\theta_{c}\right)}^{\frac{1}{2}}≜i\beta \text{,}$$

where we define *β* as the extinction coefficient of the evanescent field in the external medium, i.e.,

(17) $$\beta =\frac{\omega n_{\text{int}}}{c}{\left(\sin^{2}\;\theta_{i}-\sin^{2}\;\theta_{c}\right)}^{\frac{1}{2}}.$$

In fact, *β* is also typically defined as the propagation wavenumber of the transmitted light wave. Thus, Eq. (9) can be written as scalar form,

(18) $$E_{tz}=E_{t0}\exp\left[i\left(\beta z-\omega t\right)\right]\text{.}$$

For a general SMF, the light wave propagates down the longitudinal axis. Thus, the incident angle relative to the normal of the core/cladding surface is

(19) $$\theta_{i}\approx \frac{\pi }{2}\text{ rad.}$$

Then Eq. (17) reduces to

(20) $$\beta \approx \frac{\omega n_{\text{int}}}{c}{\left(1-\sin^{2}\;\theta_{c}\right)}^{\frac{1}{2}}=\frac{2\pi }{\lambda }{\left(n_{\text{int}}^{2}-n_{\text{ext}}^{2}\right)}^{\frac{1}{2}}\text{.}$$

Substituting it into Eq. (17),

(21) $$E_{tz}=E_{t0}\exp\left[-\frac{2\pi }{\lambda }{\left(n_{\text{int}}^{2}-n_{\text{ext}}^{2}\right)}^{\frac{1}{2}}z+i\omega t\right]\text{.}$$

Thus, the evanescent field intensity

(22) $$I_{ev}=E_{tz}E_{tz}^{*}={\left|E_{tz}\right|}^{2}=E_{t0}^{2}\exp\left[-\frac{\pi }{\lambda }{\left(n_{\mathrm{int}}^{2}-n_{\text{ext}}^{2}\right)}^{\frac{1}{2}}z\right]=I_{t0}\exp\left[-\frac{\pi }{\lambda }{\left(n_{\text{int}}^{2}-n_{\text{ext}}^{2}\right)}^{\frac{1}{2}}z\right].$$

*I*_*t*0_ is the original light intensity propagating in the waveguide. Thus, a certain portion of *I*_*t*0_ will act as an evanescent field that can indeed pierce through the internal and external boundary of the waveguiding structure, and then penetrate into the external region with decreasing amplitude, as shown in Figure 10(b). However, it has been verified that the time average of this evanescent field always vanishes [66], implying that no net lasting energy flows into the external region. Thus, the energy of the evanescent field can only flow to and fro around the boundary lying within the range of the well-known Goos–Hänchen displacement, but there is no net energy exchange between the two regions, as shown in Figure 10(c).

But it should be noted that such a loss-free regime exists only when the external region is loss-free for the evanescent field. If there is some absorbing material in the external region, only a portion of the piercing evanescent field, noting the related ratio as κ , can return and propagate through the waveguide. Considering that in most investigations only an extremely thin (typically in micrometer scale) film of externally coated material is required, for simplicity, we can assume that *I*_*ev*_ is constant across the thickness of the film, equivalent to the intensity at a certain intermediate point where we note its *z*-direction coordinate as *z*_*c*_. Thus,

(23) $$I_{ev}\left(z_{c}\right)=I_{t0}\exp\left[-\frac{\pi }{\lambda }{\left(n_{\text{int}}^{2}-n_{\text{ext}}^{2}\right)}^{\frac{1}{2}}z_{c}\right].$$

Although both *n*_in_ and *n*_ex_ are functions of intensity for high peak power pulses, the resulted deviations from their original values are rather small. Thus, for a rough evaluation, both of them can be seen as constants in Eq. (23). Then the part $\exp\left[-\frac{\pi }{\lambda }{\left(n_{\text{int}}^{2}-n_{\text{ext}}^{2}\right)}^{\frac{1}{2}}z_{c}\right]$ as a whole reduces to a constant, noting as η∈(0,1) .

(24) $$I_{ev}\left(z_{c}\right)=\eta I_{t0}$$

The transmittivity *T* of the externally coated device can be eventually written as

(25) $$T=\frac{I_{t0}-\left(1-\kappa \right)I_{ev}\left(z_{c}\right)}{I_{t0}}=1-\left(1-\kappa \right)\eta .$$

Nonlinear absorption coefficient *α* of the externally coated material can be written as

(26) $$\alpha =\frac{\alpha_{0}}{1+I_{ev}/I_{\text{sat}}}+\alpha_{ns},$$

where *α*_0_ is the modulation depth, *I*_sat_ saturable intensity, and *α*_ns_ nonsaturable absorption coefficient. As the evanescent light with an intensity of *I*_*ev*_ flows to and fro around the boundary, the induced overall loss of intensity should be

(27) $$1-\kappa =2\alpha =\frac{2\alpha_{0}}{1+\eta I_{t0}/I_{\text{sat}}}.$$

Thus,

(28) $$T=1-\frac{2\alpha_{0}\eta }{1+\eta I_{t0}/I_{\text{sat}}}=1-2\alpha_{0}{\left(\frac{1}{\eta }+\frac{I_{t0}}{I_{\text{sat}}}\right)}^{-1}$$

This directly verifies that the saturable absorbing material-coated waveguide, as a whole, can indeed function as an SA through evanescent field interaction: *T* becomes larger as *I*_*t*0_ increases.

Figure 10(d) through (f) schematically demonstrates the evanescent field interaction characteristics. When there is no absorbing material coated externally, both the guided and evanescent waves can propagate loss-freely through the waveguide, as seen in Figure 10(d). However, once there is some saturable absorbing material coated externally, there will be a portion of the evanescent wave being absorbed and then dissipating into heat. If the guided wave is weak, a considerable amount of the evanescent wave will be dissipated as heat, as shown in Figure 10(e). However, if the guided wave is stronger, the associated evanescent wave also becomes stronger, which makes the absorbing material more saturated. Thus, as seen in Figure 10(f), now the coated material becomes less absorptive to the evanescent wave, yielding less dissipated heat.

## 4 Conclusions

In conclusion, we have investigated in detail the local nonlinearities of both TF and DF embedding in BPQDs. Our results verify that engineering the local nonlinearity of a microfiber device coated with saturable absorbing material can significantly modify the mode-locking characteristics of the fiber laser. We also reveal the quite different local nonlinearities between the TF and DF devices, in both experiment and theory. In further, we reveal the general mechanism of saturable absorption with evanescent-field interactions. Our results will provide in-depth knowledge about the local nonlinearity of microfiber devices. This will be helpful for both local nonlinearity engineering of evanescent field-related devices and manipulation of nonlinear characteristics of fiber systems, especially in the design of mode-locking devices based on microfibers and nonlinearity management of ultrafast fiber lasers.
